# Lipocalin-2在肺癌患者血清中的表达及其临床意义

**DOI:** 10.3779/j.issn.1009-3419.2021.102.08

**Published:** 2021-02-20

**Authors:** 黎明 樊, 原 田, 颖 孙, 志东 胡

**Affiliations:** 300052 天津，天津医科大学总医院医学检验科 Department of Clinical Laboratory, Tianjin Medical University General Hospital, Tianjin 300052, China

**Keywords:** Lipocalin-2, 肺肿瘤, 肿瘤标志物, Lipocalin-2, Lung neoplasms, Tumor biomarker

## Abstract

**背景与目的:**

肺癌是全球发病率最高的癌症类型，严重威胁着人类健康。肺癌的早期诊断、早期治疗对于肺癌患者的生存尤为重要。血清中的肿瘤标志物作为肿瘤早期诊断的一种重要方法已被广泛应用。然而，肺癌的早期诊断标志物还很少。本研究旨在探讨Lipocalin-2在肺癌患者血清中的表达水平及其临床意义。

**方法:**

采用酶联免疫吸附法（enzyme linked immunosorbent assay, ELISA）检测Lipocalin-2在60例肺癌患者与63例健康人群外周血血清中的浓度，并分析Lipocalin-2表达水平与肺癌临床特征之间的关系。

**结果:**

Lipocalin-2在肺癌患者外周血血清中的表达水平明显高于健康人群，差异具有明显统计学意义（*P* < 0.001）。Lipocalin-2在肺癌患者中的表达与病理组织的分化、分期及淋巴结转移相关，差异具有明显统计学意义（*P* < 0.05）。Lipocalin-2在病理分化差的肺癌患者血清中的表达高于分化良好患者；在发生淋巴结转移的肺癌患者血清中的表达高于没有发生淋巴结转移患者；在临床Ⅲ期+Ⅳ期肺癌患者中的表达水平显著高于临床Ⅰ期Ⅱ期患者；差异均具有统计学意义（*P* < 0.05）。

**结论:**

Lipocalin-2在肺癌患者血清水平中高表达，与病理组织的分化、分期及淋巴结转移相关，有望成为一种潜在的用于临床诊断的新型肺癌肿瘤标志物。

## Abstract (en)

**Background and objective:**

Lung cancer is the highest incidence of cancer in the world, which seriously threatens human health. Early diagnosis and treatment of lung cancer is particularly important for the survival of lung cancer patients. Serum tumor markers have been widely used as an important method for early diagnosis of tumor. However, there are few early diagnostic markers for lung cancer. Therefore, the aim of this study was to investigate the expression level of Lipocalin-2 and its clinical significance in serum of patients with lung cancer.

**Methods:**

The serum levels of Lipocalin-2 in 60 lung cancer patients and 63 healthy people were detected by enzyme-linked immunosorbent assay (ELISA), and the relationship between the expression level of Lipocalin-2 and the clinical characteristics of lung cancer was analyzed.

**Results:**

The expression level of Lipocalin-2 in peripheral blood serum of patients with lung cancer was significantly higher than that of healthy people, and the difference was statistically significant (*P* < 0.001). The expression of Lipocalin-2 in patients with lung cancer was related to the differentiation, stage and lymph node metastasis of pathological tissues, and the difference was statistically significant (*P* < 0.05). The expression level of Lipocalin-2 in serum of patients with poorly differentiated lung cancer was higher than that of patients with well differentiated lung cancer; the expression level of Lipocalin-2 in serum of patients with lymph node metastasis was higher than that of patients without lymph node metastasis; the expression level of Lipocalin-2 in patients with clinical stage Ⅲ + Ⅳ lung cancer was significantly higher than that of patients with clinical stage Ⅰ + Ⅱ lung cancer, and the differences were statistically significant (*P* < 0.05).

**Conclusion:**

Lipocalin-2 is highly expressed in serum of patients with lung cancer, which is related to pathological differentiation, stage and lymph node metastasis. It is expected to become a potential new tumor marker for clinical diagnosis of lung cancer.

在世界范围的癌症中，肺癌是导致死亡的重要原因，其发病率、死亡率都很高，在中国，肺癌也是最常见的癌症类型^[1]^。其中，非小细胞肺癌（non-small cell lung cancer, NSCLC）占比超过83%，主要包括腺癌、鳞状细胞癌、大细胞癌等^[2]^。早期肺癌经过根治手术切除，其5年生存率最高可达80%以上，但75%的NSCLC患者诊断时已处于中晚期，治疗方式主要有手术、放化疗、靶向治疗、免疫治疗，其5年生存率低于30%^[3]^。因此，需要进一步探索肺癌的生物学特点及其分子作用机制，而寻找早期诊断肺癌的方法对于提高肺癌治愈率、改善肺癌患者预后显得尤为重要。

Lipocalin-2（LCN2），又名24p3、SIP24、NGAL，是一种小分子分泌型蛋白，最早被Hraba-Renevey等^[4]^在小鼠的肾脏细胞中发现。Lipocalin-2是Lipocalin超家族中的一员。Lipocalin-2作为一种铁转运蛋白，在多种细胞中表达，与感染性疾病、炎性肠疾病、神经退化性疾病、肥胖与代谢综合征等相关^[5]^。Lipocalin-2和肿瘤的发生发展也相关。本研究旨在通过检测Lipocalin-2在肺癌血清中的表达水平，分析其与临床病理特征的相关性。

## 1 资料与方法

### 1.1 研究对象

该研究共检测了60例肺癌患者和63例健康对照者外周血血清标本中的Lipocalin-2表达情况，肺癌患者外周血取自天津医科大学总医院2014年5月-2014年12月收治的患者。肺癌患者纳入标准：患者均为初诊并经病理学检查确诊为肺癌，且术前未行放疗、化疗、靶向药物治疗及生物治疗等。60例肺癌患者中，男性37例，女性23例，中位年龄63岁（45岁-81岁），既往有吸烟史38例，无吸烟史22例; 肿瘤最大径≤3 cm 25例， > 3 cm 35例; 腺癌40例，鳞癌20例，低分化22例，中-低分化9例，中分化18例，高分化11例，有淋巴结转移39例，无淋巴结转移21例。63例健康对照者中，男性36例，女性27例，中位年龄57岁（36岁-78岁）。本项研究通过了医院伦理委员会批准并取得患者的知情同意。

### 1.2 研究方法

采用酶联免疫吸附测定（enzyme linked immunosorbent assay, ELSIA）检测血清中的Lipocalin-2表达情况。其中ELISA试剂盒购自美国R & D公司（货号：DLCN20）。实验步骤按照试剂盒说明书操作。实验步骤如下：将所需试剂复温后，首先向每一个检测孔中加入100 µL的稀释液，然后加入标椎品、对照及实验样品，4 oC孵育2 h后洗掉孔内液体，再加入200 µL偶联试剂，4 oC孵育2 h，用洗涤液清洗后加入200 µL底物溶液，室温孵育30 min后加入终止液，最后检测吸光度（optical density, OD_450_）。将肺癌患者及健康对照者的血清，经ELISA试剂盒检测其中Lipocalin-2的表达后，通过标准样品计算各组血清中Lipocalin-2的具体浓度，然后通过比较分析各个不同类型肺癌患者血清中Lipocalin-2的含量，最后统计Lipocalin-2表达量与肺癌患者临床参数的相关性。

### 1.3 统计学方法

采用SPSS 16.0软件（Chicago, IL, USA）对数据进行统计分析，采用GraphPad Prism 8（San Diego, CA, USA）软件对数据进行作图，肺癌患者及健康人对照者血清Lipocalin-2浓度使用均数±标准差（Mean±SD）描述，统计分析采用*t*检验。当*P* < 0.05时被认为差异有统计学意义。

## 2 结果

### 2.1 Lipocalin-2在外周血中的浓度比较

Lipocalin-2在肺癌患者外周血血清中的浓度明显高于健康对照者[(204.4±81.78) ng/mL *vs* (86.83±23.13) ng/mL]，两者之间差异有显著统计学意义（*P* < 0.001）。见图 1。

**1:**
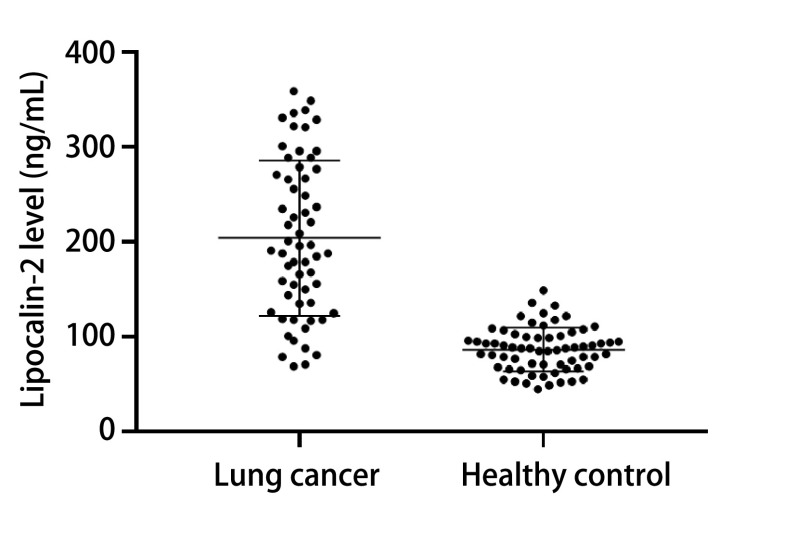
Lipocalin-2在肺癌患者及健康正常人外周血中的浓度 Lipocalin-2 level in peripheral blood of lung cancer patients and healthy people

### 2.2 Lipocalin-2在肺癌患者外周血血清中的浓度和临床病理参数之间的相关性

利用ELISA实验结果比较Lipocalin-2浓度与和肺癌患者临床病理参数的关系，结果显示，肺癌患者外周血血清Lipocalin-2浓度与患者年龄、性别、吸烟史无相关性，而与病理组织的分化、分期及淋巴结转移相关，差异具有统计学意义（*P* < 0.05）。Lipocalin-2在病理分化差的肺癌患者血清中的表达高于分化良好患者; 在发生淋巴结转移的肺癌患者血清中的表达高于没有发生淋巴结转移患者; 在临床Ⅲ期+Ⅳ期肺癌患者中的表达水平显著高于临床Ⅰ期+Ⅱ期患者，差异均具有统计学意义（*P* < 0.05）。见表 1。

**1:** 肺癌患者外周血血清中的Lipocalin-2浓度和临床病理参数之间的相关性（*n*=60） Correlation between serum Lipocalin-2 level and clinical features in patients with lung cancer (*n*=60)

Parameter	*n*	Lipocalin-2 level (ng/mL)	*P*
Age (yr)			0.361
< 60	19	211.0±85.6	
≥60	41	190.0±72.7	
Gender			0.916
Male	37	203.5±86.8	
Female	23	205.8±74.7	
Smoking history			0.568
Yes	38	199.8±85.6	
No	22	212.4±75.9	
Histological type			0.905
Adenocarcinoma	40	203.5±61.1	
Squamous cell carcinoma	20	206.2±114.4	
Tumor size			0.112
< 3 cm	25	224.3±84.9	
≥3 cm	35	190.2±77.5	
Tumor differentiation			0.040
Well	29	182.2±74.5	
Poor	31	225.2±83.8	
Lymph node metastasis			
Yes	39	237.4±74.6	< 0.001
No	21	143.2±55.5	
Clinical stage			0.001
Ⅰ+Ⅱ	33	175.3±80.1	
Ⅲ+Ⅳ	27	240.0±69.8	

## 3 讨论

Lipocalin-2（LCN2），又名中性粒细胞明胶酶相关脂质运载蛋白（neutrophil gelatinase-associated lipocalin, NGAL），在多种肿瘤组织及细胞中呈现高表达，比如肺癌、胰腺癌、结肠癌等^[6]^。在乳腺癌中，Lipocalin-2的表达和肿瘤的进展相关，在乳腺癌进展期Ⅱ期和Ⅲ期中高表达，并且在发生转移的乳腺癌患者的尿液中也呈现高表达，可用于乳腺癌患者的预测^[7]^。Lipocalin-2在高度恶性子宫内膜癌中高表达，预示着不良的总生存期及无病生存期，具有重要的预后意义^[8]^。在前列腺癌组织中，Lipocalin-2的表达和肿瘤的侵袭相关，并且和肿瘤细胞的侵袭能力相一致^[9]^。

Lipocalin-2在肿瘤中的功能主要通过以下几点实现：①通过促进肿瘤细胞的增殖、侵袭、转移促进肿瘤的进展，体内实验^[7, 10]^显示Lipocalin-2过表达的乳腺癌细胞促进了肿瘤的原位侵袭与生长; ②Lipocalin-2可调节肿瘤微环境，Lipocalin-2作为铁结合蛋白参与铁转运，Lipocalin-2介导的细胞内铁含量抑制了肿瘤细胞的凋亡^[11, 12]^; ③Lipocalin-2通过与其他蛋白的相互作用调控肿瘤的进展，在胰腺癌中MUC4通过人表皮生长因子受体-2（human epidermal growth factor receptor 2, HER2）/丝氨酸/苏氨酸蛋白激酶（serine/threonine-protein kinase, AKT）/核因子κB（nuclear factor kappa B, NF-κB）途径调节Lipocalin-2表达^[13]^。

Lipocalin-2在一些肿瘤患者血清中的表达也呈现增强趋势，其在甲状腺乳头状癌患者血浆中的含量提高，与临床参数呈现相关性^[14]^。Lipocalin-2在Ⅳ期宫颈癌患者血清中的浓度高于Ⅰ期^[15]^，但Lipocalin-2在肺癌血清中的研究较少。

本研究采用ELISA法检测Lipocalin-2在60例肺癌患者与63例健康人群外周血血清中的浓度，结果发现Lipocalin-2在肺癌患者外周血血清中的表达水平明显高于健康人群，差异具有明显统计学意义（*P* < 0.001）。Lipocalin-2在肺癌患者中的表达与病理组织的分化、分期及淋巴结转移相关，差异具有统计学意义（*P* < 0.05）。通过进一步与肺癌的病理特征比较发现，Lipocalin-2在病理分化差的肺癌患者血清中的表达高于分化良好患者; 在发生淋巴结转移的肺癌患者血清中的表达高于没有发生淋巴结转移患者; 在临床Ⅲ期+Ⅳ期肺癌患者血清中的表达水平显著高于临床Ⅰ期+Ⅱ期患者，差异均具有统计学意义（*P* < 0.05）。结果提示，Lipocalin-2可能作为一种潜在的肺癌肿瘤标志物用于临床诊断。

综上所述，Lipocalin-2在肺癌患者血清水平中高表达，其与病理组织的分化、分期及淋巴结转移相关，有望成为一种潜在的新型肺癌肿瘤标志物用于临床诊断。本研究纳入的样本量较少，不足以用于临床诊断，但为进一步大规模的检测奠定了基础，为临床应用提供了参考。
